# The establishment and validation of a prediction model for traumatic intracranial injury patients: a reliable nomogram

**DOI:** 10.3389/fneur.2023.1165020

**Published:** 2023-05-25

**Authors:** Jia Yi Chen, Guang Yong Jin, Long Huang Zeng, Bu Qing Ma, Hui Chen, Nan Yuan Gu, Kai Qiu, Fu Tian, Lu Pan, Wei Hu, Dong Cheng Liang

**Affiliations:** ^1^Department of Critical Care Medicine, Affiliated Hangzhou First People's Hospital, Zhejiang University School of Medicine, Hangzhou, China; ^2^Department of Critical Care Medicine, Hangzhou Geriatric Hospital, Hangzhou, China; ^3^Department of Intensive Care Unit, The Affiliated Hospital of Hangzhou Normal University, Hangzhou, China

**Keywords:** traumatic brain injury, mortality, risk factors, nomogram, prediction model

## Abstract

**Objective:**

Traumatic brain injury (TBI) leads to death and disability. This study developed an effective prognostic nomogram for assessing the risk factors for TBI mortality.

**Method:**

Data were extracted from an online database called “Multiparameter Intelligent Monitoring in Intensive Care IV” (MIMIC IV). The ICD code obtained data from 2,551 TBI persons (first ICU stay, >18 years old) from this database. R divided samples into 7:3 training and testing cohorts. The univariate analysis determined whether the two cohorts differed statistically in baseline data. This research used forward stepwise logistic regression after independent prognostic factors for these TBI patients. The optimal variables were selected for the model by the optimal subset method. The optimal feature subsets in pattern recognition improved the model prediction, and the minimum BIC forest of the high-dimensional mixed graph model achieved a better prediction effect. A nomogram-labeled TBI-IHM model containing these risk factors was made by nomology in State software. Least Squares OLS was used to build linear models, and then the Receiver Operating Characteristic (ROC) curve was plotted. The TBI-IHM nomogram model's validity was determined by receiver operating characteristic curves (AUCs), correction curve, Hosmer-Lemeshow test, integrated discrimination improvement (IDI), net reclassification improvement (NRI), and decision-curve analysis (DCA).

**Result:**

The eight features with a minimal BIC model were mannitol use, mechanical ventilation, vasopressor use, international normalized ratio, urea nitrogen, respiratory rate, and cerebrovascular disease. The proposed nomogram (TBI-IHM model) was the best mortality prediction model, with better discrimination and superior model fitting for severely ill TBI patients staying in ICU. The model's receiver operating characteristic curve (ROC) was the best compared to the seven other models. It might be clinically helpful for doctors to make clinical decisions.

**Conclusion:**

The proposed nomogram (TBI-IHM model) has significant potential as a clinical utility in predicting mortality in TBI patients.

## 1. Introduction

Traumatic brain injury (TBI) is “an alteration in brain function caused by an external force” (1, 2). Epidemiological data from developed countries have changed in the past few years. TBI is a critical public health problem worldwide that can cause death and disability. Approximately 64–74 million people sustain TBI annually (3). Mild and severe TBI affects ~55.9 million and 5.48 million individuals, respectively (4). Furthermore, TBI is estimated to contribute to a third of all US injury-related deaths (30.5%) (5). A deterministic linkage dataset study in 2016 found that falling was Belgium's predominant cause of injury. Road safety and aging patients with TBI have increased recently, reducing transport-related TBI mortality and causing a shift in the health care system (1, 6, 7). Over the past two decades, significant advancements have been made in managing severe TBI both surgically and medically (8). The prognosis of patients with TBI is poor. TBI typically results in physical disability, cognitive dysfunction, and mortality, increasing direct and indirect medical costs. TBI also impacts systemic inflammation. Numerous secondary injuries, such as multiple molecular and activated cellular pathways develop as the primary injury progresses (2).

This study investigated if mortality can be lowered by increasing awareness and focusing interventions on patients with TBI to improve patient outcomes. It is essential to block reversible factors by studying death-related factors as soon as possible to upturn TBI patients' mortality and modify their prognosis and outcome. Some predictive models have been developed for TBI subgroups. These include subarachnoid hemorrhage, traumatic brain parenchyma hematoma, and moderate or severe pediatric TBI patients who undergo a second operation (9–12). However, it is challenging to predict the outcome of patients with TBI. Several medical problems have been predicted using nomograms, a two-dimensional (2D) calculator developed from a mathematical function (9). As a result of individual prognosis predictions, clinicians can make better clinical decisions, decreasing mortality rates.

This retrospective study analyzed the clinical data of 2,551 patients with TBI from MIMIC IV. In addition, risk factors for TBI were explored, which were at hospital admission. In addition, if we could explore the TBI mortality at patients' hospital admission, timely intervention can improve outcomes. The final objective of this model was to reduce the mortality of TBI.

## 2. Materials and methods

### 2.1. Study design and participants

**Figure 1:**
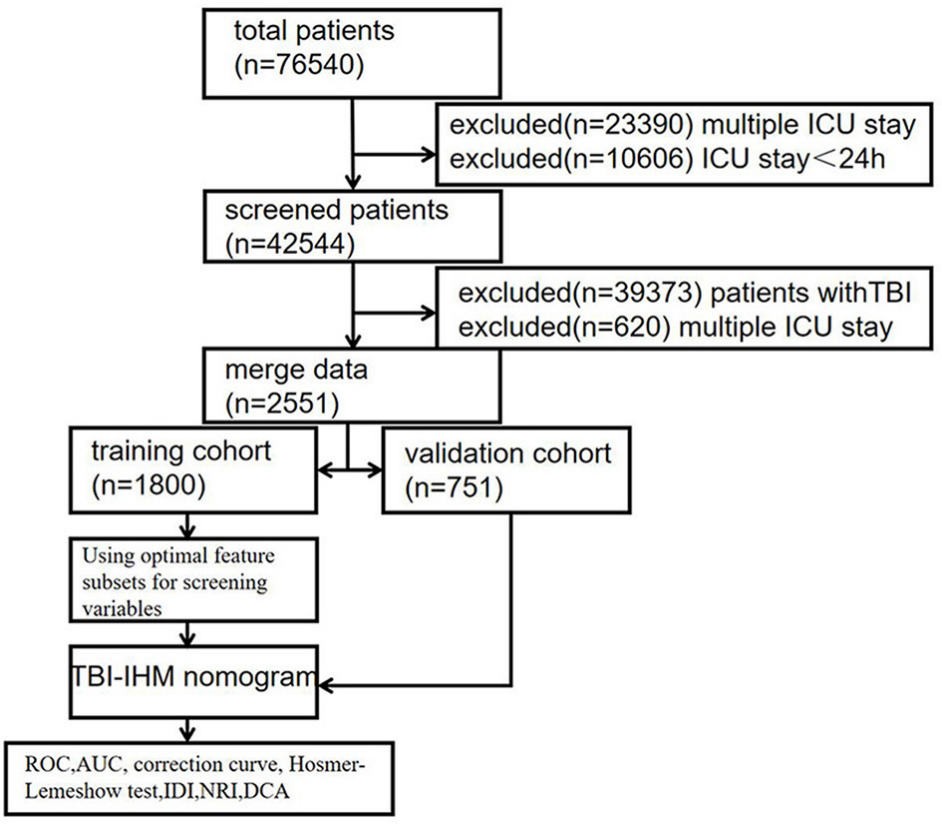
The enrollment flowchart of the study population in the training and validation cohorts.

Several limitations exist in identifying the risk of lethal TBI. Therefore, this research was based on the baseline characteristics and clinical characteristics data of TBI patients from the Multiparameter Intelligent Monitoring in Intensive Care IV (MIMIC IV) database. The Laboratory for Computational Physiology at the Massachusetts Institute of Technology maintained the database. It contained information on more than 30,000 patients in the ICU at Beth Israel Deaconess Medical Center from 2008 to 2019. The participants signed consent to complete the training course (CITI Data or Specimens Only Research, PhysioNet Credentialed Health Data Use Agreement) of the National Institutes of Health on the Internet. The dataset comprised 2,551 patients who suffered from TBI. Collected data included clinical characteristics, treatments, and outcomes. The data were merged with State software, and the samples were randomly divided into training and testing cohorts in a 7:3 ratio using R software. The univariate analysis determined if the two cohorts had statistically different baseline data to ensure the two groups were comparable. The R's optimal subset method selected the optimal variables for the model. The computer algorithm independently filtered the risk factors of TBI patients' hospital mortality. Finally, a nomogram prediction model, TBI-IHM, was generated using the nomolog function from the State software. The optimal feature subsets in pattern recognition improved the model's prediction. The minimum BIC forest of the high-dimensional mixed-graph model achieved a better prediction effect. Consequently, a nomogram containing these risk factors was made to predict the mortality incidence for TBI patients by nomolog in State software (TBI-IHM model). This model can facilitate physicians to modify decisions regarding patients with serious traumatic intracranial injury (TBI). Least squares OLS was used to build linear models, and the receiver operating characteristic (ROC) curve was plotted. The TBI-IHM nomogram model's validity was determined by multiple indicators, including the area under the receiver operating characteristic curves (AUCs), correction curve, Hosmer-Lemeshow test, integrated discrimination improvement (IDI), net reclassification improvement (NRI), and decision-curve analysis (DCA). The design flow is shown in Figure 1.

### 2.2. Inclusion and exclusion criteria

This study excluded pregnant women and patients <18 years old. Only details of the first hospitalization were chosen for patients who had been to the ICU multiple times. The following information was extracted: age, median, women, BMI, admission type, marital status (single, married, urgent, and other), ethnicity (Black, white, Asian, and Other), first care unit (trauma SICU, neurosurgical intensive care unit, medical intensive care unit, surgical intensive care unit, and other), underlying diseases (hypertension, myocardial infarction, congestive heart failure, peripheral vascular disease, cerebrovascular disease, chronic pulmonary disease, liver disease, diabetes, paraplegia, renal disease, and metastatic solid tumor), Charlson Comorbidity Index, and disease severity (First day GCS, Firstday APS-III, Firstday SOFA, Firstday LODS, Firstday SAPS-II, and Firstday OASIS) (Table 1). In addition, the following information was also extracted: vital signs (temperature, heart rate, respiratory rate, MAP, SPO2, and glucose), blood routine test (white blood cells, hemoglobin, hematocrit, and platelets), biochemical indicators (creatinine, urea nitrogen, blood sodium, blood potassium, blood calcium, blood magnesium, blood chloride, blood phosphate, prothrombin time, partial thromboplastin time, INR, bicarbonate, and anion gap), interventions within 24 h of ICU admission (use of vasopressor, use of albumin, use of furosemide, use of mannitol, use of MV, and use of RRT), and outcomes (in-hospital mortality, ICU mortality, and hospital LOS) (Table 2). All eligible patients were divided into two groups (according to the in-hospital death or not).

**Table 1:** The baseline characteristics of all participants according to the training or validation cohort.

**Characteristic**	**Whole population (*N* = 2,551)**	**Training cohort (*N* = 1,800)**	**Validation cohort (*N* = 751)**	***P*-value**
Age, median (IQR)	65.69 (48.53–80.23)	66.00 (49.03–80.30)	64.73 (47.57–79.95)	0.638
Female, No. (%)	1,000 (39.2)	704 (39.1)	296 (39.4)	0.886
BMI, median (IQR) (kg/m^2^)	25.77 (22.43–29.78)	25.73 (22.40–29.69)	25.82 (22.53–30.04)	0.503
**Admission type, No. (%)**				0.551
Emergency	1,739 (68.2)	1,225 (68.1)	514 (68.4)	
Observation	513 (20.1)	366 (20.3)	147 (19.6)	
Urgent	213 (8.4)	144 (8.0)	69 (9.2)	
Other	86 (3.4)	65 (3.6)	21 (2.8)	
**Marital status, No. (%)**				0.200
Single	700 (27.4)	473 (26.3)	227 (30.2)	
Married	940 (36.9)	673 (37.4)	267 (35.6)	
Divorced	128 (5.0)	93 (5.2)	35 (4.7)	
Widowed	292 (11.5)	201 (11.2)	91 (12.1)	
Other	491 (19.3)	360 (20.0)	131 (17.4)	
**Ethnicity, No. (%)**				0.663
Black	162 (6.4)	119 (6.6)	43 (5.7)	
White	1,528 (59.9)	1,071 (59.5)	457 (60.9)	
Asian	69 (2.7)	52 (2.9)	17 (2.3)	
Other	792 (31.1)	558 (31.0)	234 (31.2)	
**First care unit, No. (%)**				0.901
Trauma SICU (TSICU)	1,082 (42.4)	759 (42.2)	323 (43.0)	
Neuro surgical intensive care unit	291 (11.4)	206 (11.4)	85 (11.3)	
Surgical intensive care unit (SICU)	589 (23.1)	417 (23.2)	172 (22.9)	
Medical intensive care unit (MICU)	133 (5.2)	90 (5.0)	43 (5.7)	
Other	456 (17.9)	328 (18.2)	128 (17.0)	
**Underlying diseases, No. (%)**				
Hypertension	572 (22.4)	419 (23.3)	153 (20.4)	0.109
Myocardial infarction	187 (7.3)	138 (7.7)	49 (6.5)	0.313
Congestive heart failure	289 (11.3)	204 (11.3)	85 (11.3)	0.991
Peripheral vascular disease	131 (5.1)	91 (5.1)	40 (5.3)	0.778
Cerebrovascular disease	517 (20.3)	384 (21.3)	133 (17.7)	0.038
Chronic pulmonary disease	285 (11.2)	205 (11.4)	80 (10.7)	0.590
Liver disease	141 (5.5)	99 (5.5)	42 (5.6)	0.926
Diabetes	480 (18.8)	345 (19.2)	135 (18.0)	0.483
Paraplegia	266 (10.4)	200 (11.1)	66 (8.8)	0.080
Renal disease	229 (9.0)	157 (8.7)	72 (9.6)	0.486
Metastatic solid tumor	63 (2.5)	43 (2.4)	20 (2.7)	0.684
Charlson comorbidity index	4 (2–6)	4 (2–6)	4 (2–6)	0.457
**Disease severity (median, IQR)**				
First-day GCS	13 (9–14)	13 (9–14)	13 (8–14)	0.991
First-day APS-III	39 (28–54)	39 (28–54)	38 (28–53)	0.420
First-day SOFA	4 (2–6)	4 (2–6)	4 (2–6)	0.802
First-day LODS	3 (2–6)	4 (2–6)	3 (2–6)	0.190
First-day SAPS-II	31 (24–39)	31 (24–39)	31 (23–39)	0.385
First-day OASIS	32 (26–39)	32 (26–39)	32 (26–38)	0.441

**Table 2:** All participant's clinical characteristics within 24 h of ICU admission according to the training or validation cohort.

**Characteristic**	**Whole population (*N* = 2,551)**	**Training cohort (*N* = 1,800)**	**Validation cohort (*N* = 751)**	***P*-value**
**Vital Signs, median (IQR)**				
Temperature (°C)	36.56 (36.33–36.78)	36.56 (36.33–36.78)	36.56 (36.33–36.83)	0.114
Heart rate (bpm)	65.00 (57.00–75.00)	65.00 (57.00–75.00)	65.00 (57.00–75.00)	0.670
Respiratory rate (bpm)	12.00 (10.00–14.00)	12.00 (10.00–14.00)	12.00 (10.00–14.00)	0.944
MAP (mmHg)	63.00 (55.00–71.00)	63.00 (55.00–71.00)	63.00 (55.00–70.00)	0.966
SPO2 (%)	94.00 (92.00–96.00)	94.00 (92.00–96.00)	94.00 (92.00–96.00)	0.846
Glucose (mg/dL)	109.00 (93.00–127.00)	109.00 (93.00–127.00)	111.00 (94.00–127.00)	0.294
**Blood routine test (IQR)**				
White blood cells (K/μL)	9.20 (7.00–12.00)	9.30 (7.00–12.00)	9.00 (7.00–12.10)	0.502
Hemoglobin (g/dL)	11.20 (9.70–12.60)	11.20 (9.70–12.50)	11.30 (9.70–12.70)	0.597
Hematocrit (%)	33.40 (28.80–37.30)	33.40 (28.85–37.20)	33.70 (28.60–37.40)	0.892
Platelets (K/μL)	181.00 (138.00–227.00)	181.00 (138.00–229.50)	182.00 (139.00–226.00)	0.715
**Biochemical indicators (IQR)**				
Creatinine (mg/dL)	0.80 (0.60–1.00)	0.80 (0.60–1.00)	0.80 (0.70–1.00)	0.592
Urea nitrogen (mg/dL)	14.00 (10.00–19.00)	14.00 (10.00–19.00)	14.00 (10.00–19.00)	0.357
Blood sodium (mEq/L)	138.00 (136.00–141.00)	138.00 (136.00–141.00)	139.00 (136.00–141.00)	0.479
Blood potassium (mEq/L)	3.80 (3.50–4.10)	3.80 (3.50–4.10)	3.80 (3.50–4.10)	0.667
Blood calcium (mEq/L)	8.40 (7.80–8.80)	8.40 (7.80–8.80)	8.40 (7.80–8.80)	0.600
Blood magnesium (mEq/L)	1.80 (1.60–2.00)	1.80 (1.60–2.00)	1.80 (1.60–2.00)	0.146
Blood chloride (mEq/L)	103.00 (100.00–106.00)	103.00 (100.00–106.00)	103.00 (100.00–106.00)	0.970
Blood phosphate (mEq/L)	3.00 (2.50–3.60)	3.10 (2.50–3.60)	3.00 (2.50–3.50)	0.030
Prothrombin time (s)	12.20 (11.30–13.40)	12.30 (11.30–13.40)	12.10 (11.30–13.20)	0.140
Partial thromboplastin time (s)	26.40 (24.30–29.00)	26.50 (24.30–29.10)	26.20 (24.10–28.80)	0.207
INR	1.10 (1.00–1.20)	1.10 (1.00–1.20)	1.10 (1.00–1.20)	0.107
Bicarbonate (mEq/L)	22.00 (20.00–24.00)	22.00 (20.00–24.00)	22.00 (20.00–24.00)	0.765
Aniongap (mEq/L)	13.00 (11.00–15.00)	13.00 (11.00–15.00)	13.00 (12.00–15.00)	0.530
**Interventions within 24 h of ICU admission**				
Use of vasopressor (%)	383 (15.0)	278 (15.4)	105 (14.0)	0.346
Use of albumin (%)	93 (3.7)	73 (4.1)	20 (2.7)	0.087
Use of furosemide (%)	190 (7.5)	121 (6.7)	69 (9.2)	0.031
Use of mannitol (%)	203 (8.0)	139 (7.7)	64 (8.5)	0.496
Use of MV (%)	1,152 (45.2)	819 (45.5)	333 (44.3)	0.592
Use of RRT (%)	18 (0.7)	13 (0.7)	5 (0.7)	0.877
**Outcomes**				
In-hospital mortality (%)	413 (16.2)	291 (16.2)	122 (16.3)	0.961
ICU mortality (%)	312 (12.2)	221 (12.3)	91 (12.1)	0.910
Hospital LOS (days)	7.25 (4.02–13.67)	7.30 (4.03–13.71)	6.93 (4.00–13.37)	0.715
ICU LOS (days)	2.93 (1.77–6.04)	2.94 (1.77–6.03)	2.90 (1.76–6.13)	0.770

NE, anephrine; RRT, renal replacement therapy; MV, mechanical ventilation; LOS, length of stay in hospital; vasopressor_use = NE + E + dopamine + dobutamine + phenylephrine + vasopressin.

A minimum value for the continuous variable was chosen.

### 2.3. Statistical analysis

Continuous variables in the tables from this study were presented as the mean with SD or median with interquartile ranges. The student's *t*-test, the Wilcoxon rank-sum test, and the Kruskal Wallis test were used in these cases. A percentage presented categorical variables and we compared them in the *X*^2^ test. The R software (version 4.0.3) and SPSS software (version 24.0) conducted statistical analyses. Statistically significant meant *P* < 0.05. The research aimed to obtain a minimum BIC forest of the high-dimensional mixed graph model to achieve better prediction performance. The data was divided into two cohorts by random sampling: the nomogram training cohort (70%) and the performance testing cohort (30%). The least squares OLS method was used to build linear models. Then, ROC curves were plotted based on the receiver operating characteristics. This study conducted tests to identify the validity of the proposed nomogram model (TBI-IHM nomogram). These tests included net reclassification improvement (NRI), decision-curve analysis (DCA), the area under the receiver operating characteristic curve (AUC), correction curve, Hosmer-Lemeshow test, and integrated discrimination improvement (IDI).

## 3. Results

### 3.1. Training and validation cohorts' characteristics

According to the inclusion criteria, this study screened 2,551 patients from the MIMIC IV. Of these, 1,800 patients were selected as the training cohort and 751 as the validation cohort. The baseline characteristics of the training cohort and validation cohorts and the clinical characteristics of all two cohorts are listed in Tables 1, 2, respectively. The general baseline characteristics and most basic diseases were not statistically significantly different except for cerebrovascular disease. There are no statistically significant differences between the two cohorts regarding the disease severity and the scores of the scoring table related to disease severity.

### 3.2. Nomogram construction

**Figure 2:**
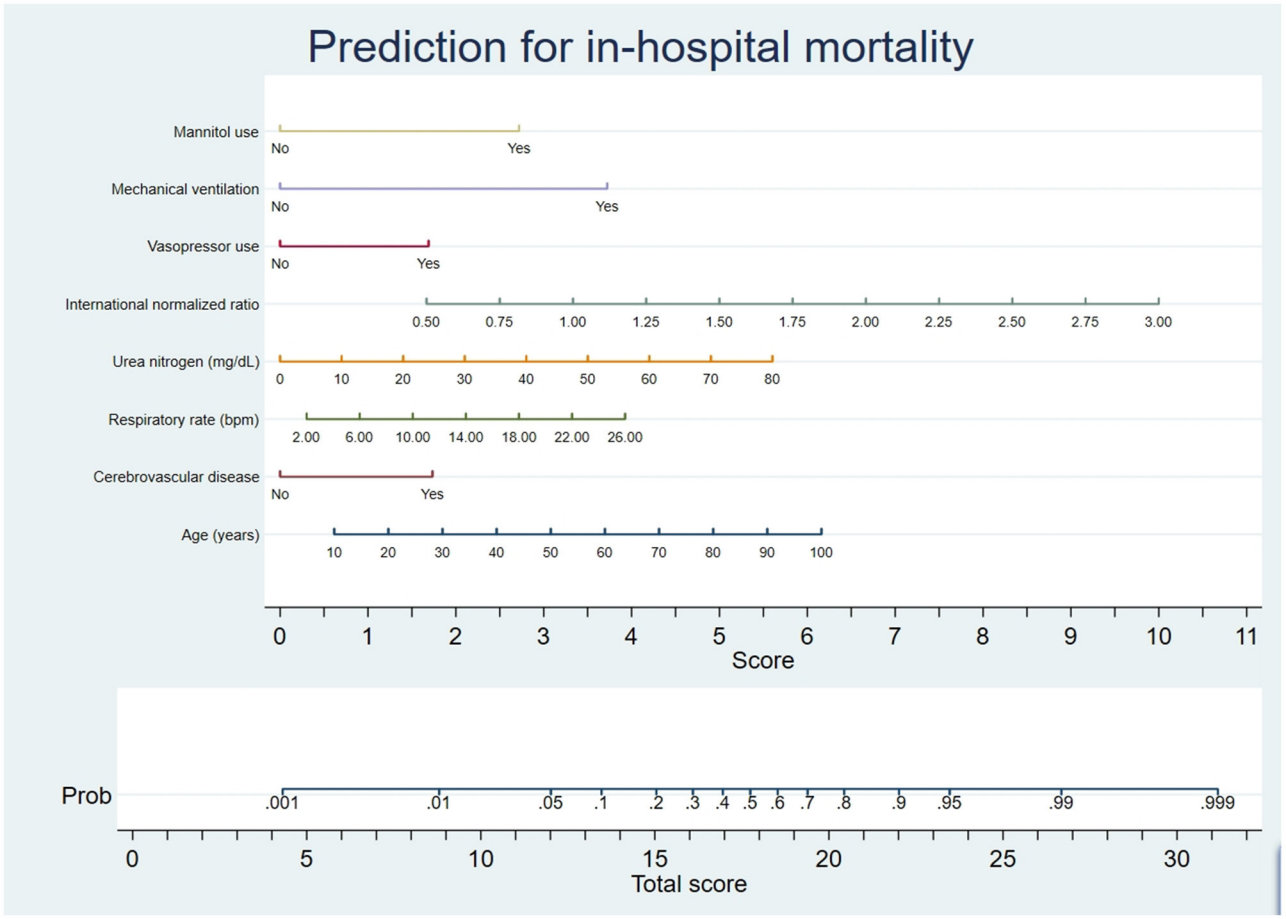
Nomogram prediction of the probability of in-hospital mortality in TBI patients. A vertical line was drawn from each variable upward to the terms on the nomogram, and the corresponding points were recorded. The point of each variable was summed up to obtain a total score corresponding to a predicted mortality probability at the nomogram's bottom.

This research used optimal feature subsets in pattern recognition to improve the model prediction. The minimum BIC forest of the high-dimensional mixed-graph model achieved a better prediction effect. The factors incorporated into the nomogram included mannitol use, mechanical ventilation, vasopressor use, international normalized ratio, urea nitrogen, respiratory rate, cerebrovascular disease, and age (Figure 2). The nomogram predicted the mortality of TBI patients by first identifying every variable position to every corresponding point on the nomogram's axis. Second, points of all variables were added to obtain a total score. Then the total score estimated the mortality probability for every patient.

### 3.3. Comparison of predictive performance between TBI-IHM nomogram and the other common clinical prognosis evaluation tables

#### 3.3.1. The training cohort

**Figure 3:**
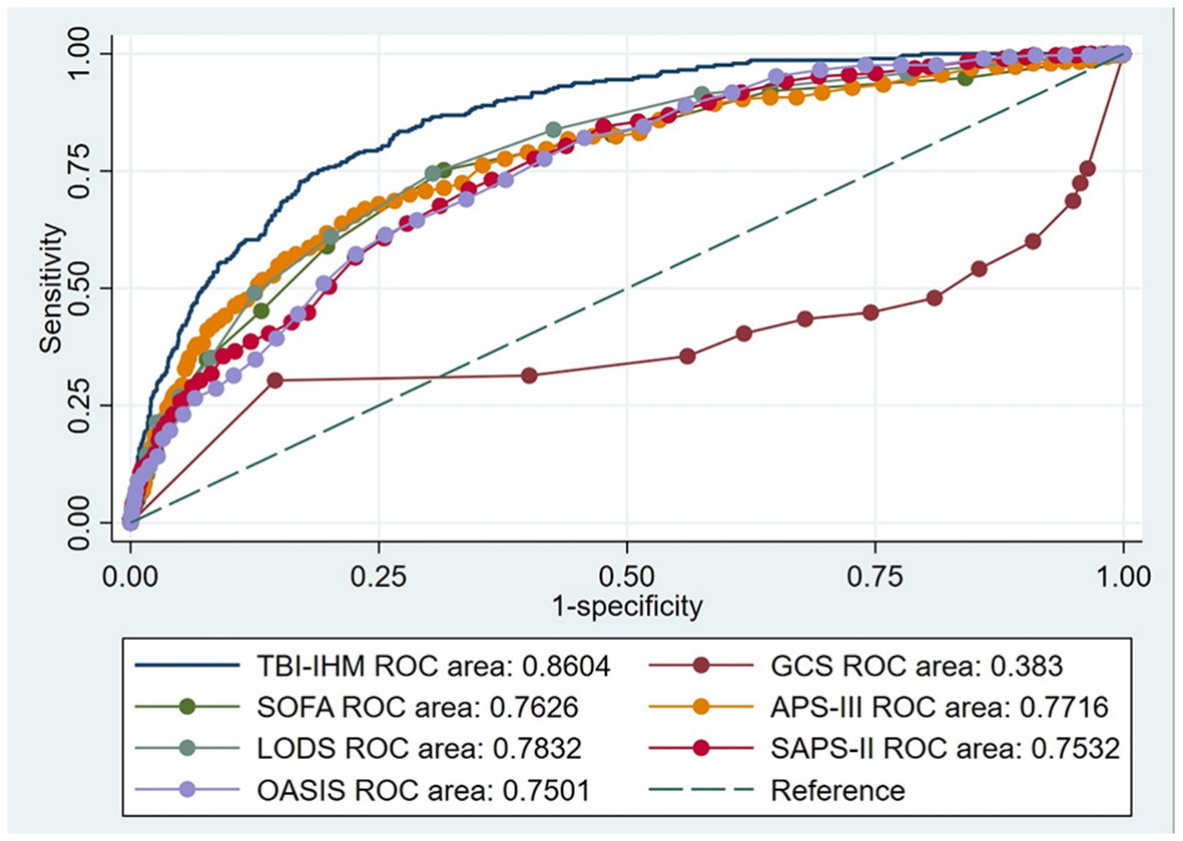
The ROCs of the TBI-IHM training cohort and the other common clinical prognosis evaluation scales. Additionally, AUCs determined that values ≥0.9 are “excellent”, ≥0.80 are “good”, ≥0.70 are “fair”, and <0.70 are “poor”.

**Figure 4:**
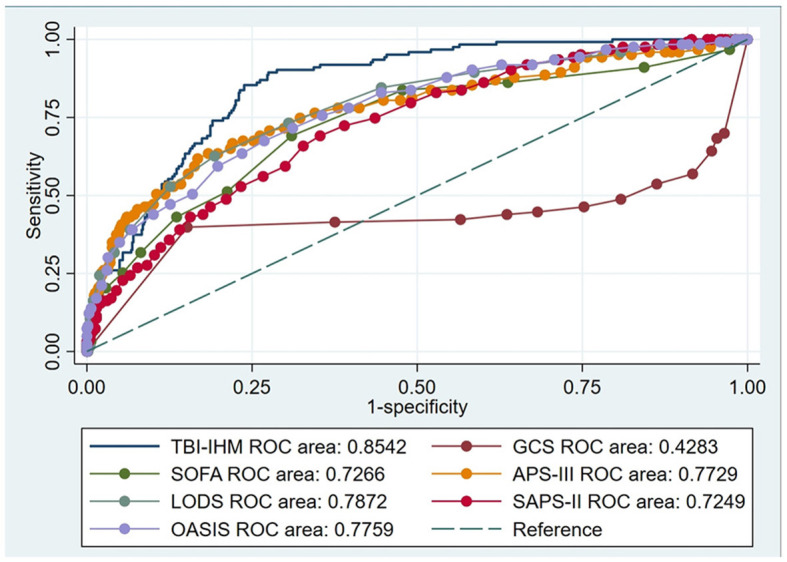
The ROCs of the TBI-IHM validation cohort and the other common clinical prognosis evaluation tables.

This research produced AUCs to estimate the discrimination of the TBI-IHM nomogram and the other six common score tables in predicting the mortality of TBI. Both nomograms in the training and the validation datasets performed better discrimination than other common score tables. AUCs of nomogram (TBI-IHM) at hospital mortality was 0.864, while the AUCs of other evaluation tables were 0.3830 (GCS), 0.7626 (SOFA), 0.7716 (APS-III), 0.7832 (LODS), 0.7532 (SAPS-II), and 0.7501 (OASIS) (Figure 3). In the validation datasets, the AUCs of TBI-IHM nomogram of hospital mortality were 0.8542, while the AUCs of other evaluation tables were 0.4283 (GCS), 0.7266 (SOFA), 0.7729 (APS-III), 0.7828 (LODS), 0.7249 (SAPS-II), and 0.7759 (OASIS) (Figure 4). The calibration curves of Nomogram1 and Nomogram2 represented higher homogeneity between the probabilities of the predicted survival and actual survival proportion than the other six common mortality forecast score tables.

**Figure 5:**
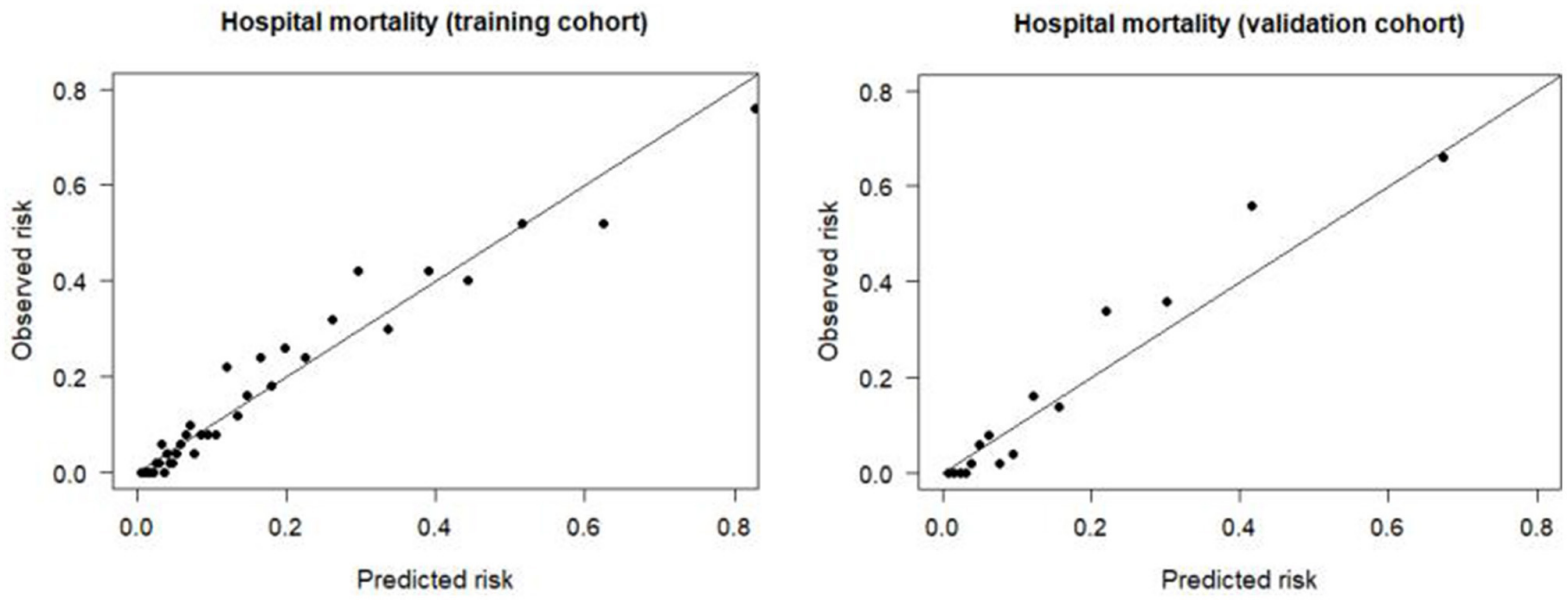
The calibration curves for the validation cohort and the training cohort.

The calibration curves of the two nomograms (Figure 5) showed that both calibration curves of the two cohorts (training and validation datasets) were almost diagonal. The Hosmer-Lemeshow test results indicated the absence of statistical significance. The training cohort: χ^2^ = 29.019, *p* = 0.7103, and the validation cohort: χ^2^ = 19.048, *p* = 0.1216, indicated that the TBI-IHM nomogram was a good fit for the data.

**Figure 6:**
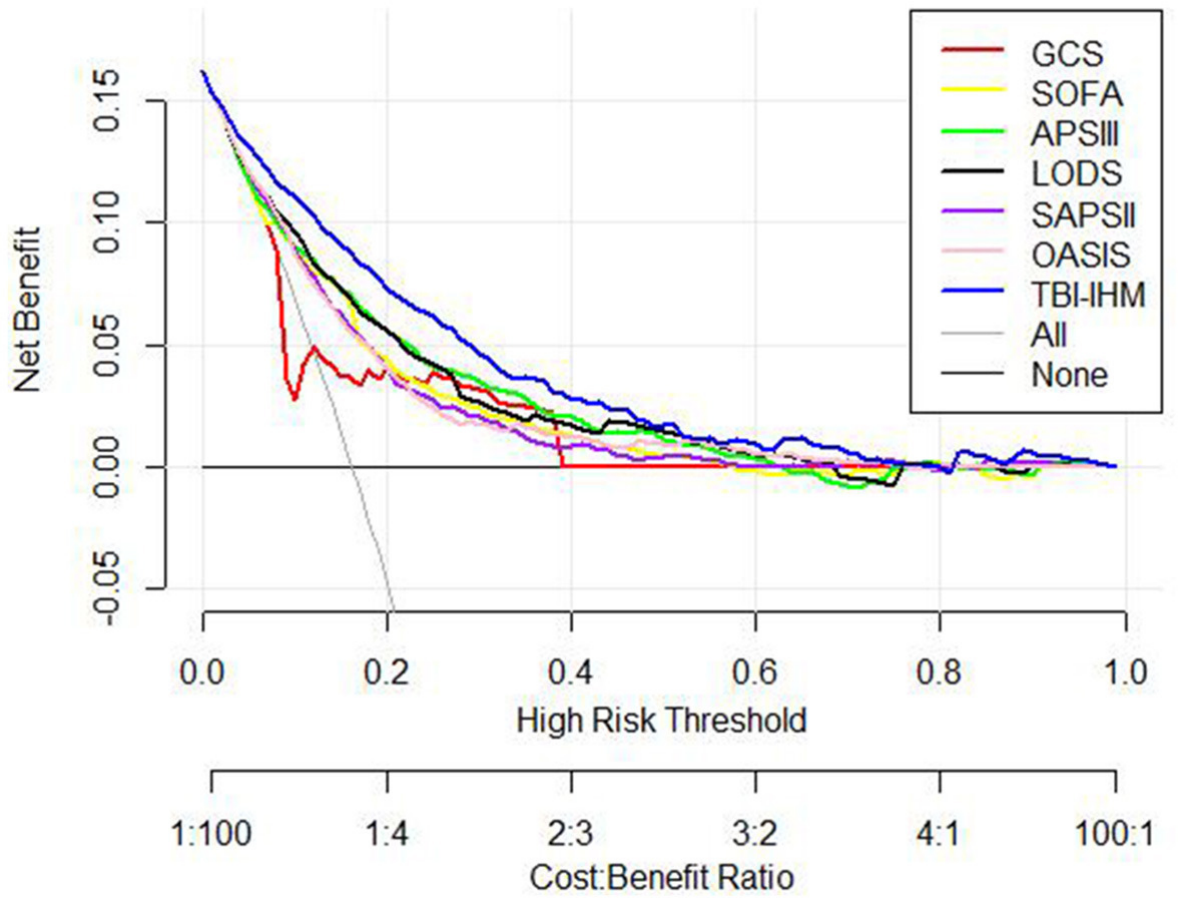
The decision-curve analysis of the validation cohort and the training cohort.

The nomogram illustrated by a DCA curve (Figure 6) displayed its clinical value. The proposed nomogram can guide some clinical interventions with a higher benefit than other common scoring scales in both cohorts. NRI and IDI of the nomogram and critical care scoring system alone in survival prediction for TBI patients are listed in Table 3.

**Table 3:** NRI and IDI of the nomogram and critical care scoring system alone in survival prediction for TBI patients.

	**Training cohort**			**Validation cohort**		
**Index**	**Estimate**	**95% CI**	***P*** **-value**	**Estimate**	**95% CI**	***P*** **-value**
NRI (vs. GCS)	0.360	0.267–0.452	<0.001	0.252	0.107–0.396	<0.001
NRI (vs. SOFA)	0.215	0.122–0.308	<0.001	0.183	0.036–0.329	0.014
NRI (vs. APS-III)	0.110	0.013–0.206	0.026	0.158	0.016–0.300	0.029
NRI (vs. LODS)	0.163	0.072–0.254	<0.001	0.178	0.043–0.313	0.010
NRI (vs. SAPS-II)	0.291	0.206–0.377	<0.001	0.310	0.169–0.450	<0.001
NRI (vs. OASIS)	0.256	0.164–0.348	<0.001	0.168	0.027–0.308	0.020
IDI (vs. GCS)	0.189	0.156–0.222	<0.001	0.173	0.125–0.221	<0.001
IDI (vs. SOFA)	0.131	0.009–0.162	<0.001	0.123	0.075–0.172	<0.001
IDI (vs. APS-III)	0.090	0.054–0.127	<0.001	0.085	0.033–0.136	0.001
IDI (vs. LODS)	0.089	0.055–0.123	<0.001	0.074	0.022–0.126	0.005
IDI (vs. SAPS-II)	0.138	0.107–0.169	<0.001	0.134	0.088–0.181	<0.001
IDI (vs. OASIS)	0.135	0.104–0.166	<0.001	0.104	0.057–0.151	<0.001

## 4. Discussion

This study aimed to develop simple and more advanced predictive models for mortality in traumatic brain injury (TBI). Nomograms are well-documented clinical prediction tools (9). A clinical examination and test can help predict mortality following a traumatic brain injury, which might help detect diseases early, accurately, and in a timely manner. This study investigated risk factors to promote better clinical decision-making based on predictive models. Nomograms were developed and validated using baseline data and laboratory examinations of patients with TBI, which can predict mortality due to TBI based on risk factors. Several validity indicators were applied to the proposed nomogram model, including the AUC, Hosmer-Lemeshow test, correction curve, NRI, IDI, and DCA. In the goodness-of-fit test (NRI, IDI), it was concluded that the calibration for the validation and development of the model was good. Therefore, mortality can be predicted more accurately with the proposed model. The use of mannitol, mechanical ventilation, vapor pressure support use, international normalized ratio, urea nitrogen, respiration rate, and cerebrovascular disease was significantly associated with age in this study.

Cerebral edema can develop due to the blood-brain barrier (BBB) disruption, inflammation of the local area, vascular abnormalities, or altered cellular metabolism of the brain. Intracranial pressure (ICP) has been reduced with the use of mannitol by removing water from cells (osmotic effect) which lowered intracranial pressure (11–13). However, excessive dehydration might impair the brain functions, such as cognitive function and mental performance, caused by hormonal dysfunctions, mitochondrial disorders, and brain cytokine elevations (14). According to Halinder S Mangat's study, hypertonic saline reduces intracranial pressure more effectively than mannitol as cerebral perfusion pressure burdens (8). The concentration of accumulated mannitol increases with extended mannitol use over time and cumulative dose, adversely affecting the osmotic gradient and reducing the therapeutic effect of intracranial pressure. Based on a meta-analysis, hypertonic saline performed better than mannitol in several experiments that involved lowering intracranial pressure and increasing cerebral perfusion (8). Compared with other intracranial pressure-lowering agents, hypertonic saline has superior efficacy and safety in TBI patients. Chen et al. collected and analyzed the RCT data of acute TBI patients with any severity randomized into RCTs. The hypertonic saline was compared with other treatments to lower intracranial pressure in the long term (13).

Aaron M. Cook's study of the TBI subtype found that hyperosmolar therapy could help reduce ICP elevations and cerebral edema caused by TBI; however, no differences were observed in neurological outcomes (15). The present research concurred with these recommendations in treating elevated intracranial pressure with hyperosmolar fluids. In some cases, mannitol usage might lead to an increase in mortality among TBI patients. According to this study's nomogram, mannitol use was associated with poor patient outcomes. Mannitol's internal pressure might increase in later use due to its increased internal pressure. It is necessary to conduct a further randomized controlled trial (RCT) study to confirm the findings. However, mannitol might be useful in TBI at some point. If the increased intracranial pressure is not reduced by mannitol, other solutions would need to be explored. Furthermore, in some TBI-related studies, early mannitol use independently increased the incidence of AKI (12). In patients with brain injuries, mannitol is associated with hypovolemia, hypotension, and increased mortality (16).

Approximately 3.9–23% of TBI patients develop AKI, which is closely related to their mortality, long-term outcome, and length and hospital stay expenses (17). Multiple mechanisms including massive catecholamine release and inflammatory mediators cause AKI after TBI. Ruoran Wang et al. indicated that AKI is most likely to occur in the first 3 days of admission of TBI patients. Their study showed that the occurrence and level of AKI at its highest point were associated with mortality, while the duration and burden of AKI were not related to mortality (18). Shuo And's study found that diuretics (furosemide torasemide), GCS score, coronary heart disease, hypertension, and vasoactive drugs (dopamine and norepinephrine) were the risk factors of AKI of TBI patients in the neural-critical care unit (19). Induced hypoperfusion by multiple causes is the leading cause of AKI development in the special early phase and the elderly phase. Systemic inflammation caused by the initial release of catecholamine and neuroinflammation due to brain injury (14) is the leading cause of AKI (17). Catecholamine surges after TBIs cause excessive activation of the renin–angiotensin–aldosterone system, leading to renal dysfunction (20). Several pathophysiological processes accompany injury in the early stage. These include hypoperfusion caused by massive bleeding, systemic inflammation for severe brain damage, and autoimmune complications. Rapid and large doses of hyperosmotic drugs administered due to these causes result in AKI patients having a poor prognosis after TBI (8, 21). Unlike mannitol, which has potentially severe side effects on the kidneys, more glycerol fructose and hypertonic saline should be used to reduce intracranial pressure.

Respiration rate and mechanical ventilation use were associated with mortality in the proposed TBI-IHM nomogram. Compared to patients who did not have hospital-acquired pneumonia, those with hospital-acquired pneumonia had worse outcomes and experienced elevated intracranial pressure (4). There was a significant association between ventilator-associated pneumonia, prolonged ICU stays, and mechanical ventilation durations (22). A prospective observational study by Chiara Robba et al. included TBI ICU patients from multi centers in Europe and found an association between an increased risk of VAP and a high risk for respiratory failure in the ICU. Other risk factors include age, younger alcohol abuse, drug abuse, thoracic trauma, chest trauma, histamine-receptor antagonist intake, and antibiotic prophylaxis (23). Compared to non-trauma patients, intubated patients in ICU with trauma had a four times higher VAP incidence rate (22).

TBI patients require regular mechanical ventilation to prevent airway obstruction and exacerbation of injuries. Most TBI patients have multiple pulmonary complications (such as pneumonia and pulmonary edema); therefore, adjusting and modulating oxygenation and ventilation is challenging. However, lowering the respiratory rate does not lead to safer outcomes. Computer algorithms were used in the present research to calculate respiratory rate parameters. In clinical prognosis, it will serve as a suggestive system that indicates that the lower the respiratory rate the better the outcome. Hypoxia or abnormal carbon dioxide retention caused by low respiratory rates may not be conducive to treating TBI patients (24, 25). Administering proper sedation could result in shorter ICU stays, fewer ventilator days (26), reduced oxygen consumption, lower respiratory rate and reduce brain oxygen consumption of patients. The proper use of sedatives can be premised on minimizing side effects by keeping sedation at lighter levels (Richmond Agitation Sedation Scale, RASS −2 to +1).

According to this study's findings, there is an increased probability of poor prognosis (mortality) in patients with abnormal INR results. TBI patients are reported to suffer from occult coagulopathy, dramatically increasing their mortality rates. Clinical outcomes are adversely affected by trauma-induced coagulopathy and secondary brain injury (27). Various mechanisms (platelet dysfunction and hyperfibrinolysis caused by inflammation, endothelial cell activation, etc.) lead to coagulopathy after TBI platelet dysfunction and continuous bleeding (28). A previous literature's statistical analysis estimates that approximately one-third of TBI patients developed coagulopathy. Abundant data cite the incidence rate of coagulopathy to be 7–63% (29). The strong association between TBI and coagulopathy is a well-recognized risk factor for poor clinical outcomes following a TBI (29). In addition to hemodynamic alterations and systemic inflammation, patients who sustain TBI show signs of these conditions (30), but their pathogenesis remains poorly understood. The research results from the literature were consistent with the present study. Consequently, it is essential to prevent traumatic coagulation disease and detect abnormal coagulation functions as soon as possible in the clinical treatment process. A timely adjustment of intervention and treatment may be necessary in cases of abnormal coagulation. An example would be the infusion of blood plasma or the administration of vitamin K.

The cerebrovascular disease has a high mortality rate because of its acute progression, quickly deteriorating into severe complications, such as brain cell edema (31). With poor prognosis and high morbidity, cerebrovascular disease patients suffer from poor quality of life and increased public health burden. Severe TBI patients need a systematic scale to guide the systematic management and avoid secondary injury (including hypotension, hypoxia, and hypoglycemia) (21, 23).

Researchers found that ~50% of severe TBI patients have suffered from infections during the hospital stay period, which might be related to the impaired immune function of patients. Some studies found that brain vasoconstrictor factors in TBI patients varied. ECF cytokine content in the brain was prominently different from jugular and arterial blood (32, 33). Lassarén et al.'s clinical trial found that the development of a systemic clinical infection led to the decrease of brain-ECF (IL1-ra, G-CSF, PDGF-ABBB, and MIP-1b) (32). However, further research is needed to investigate whether the neuroinflammatory reaction of systemic inflammation conditions causes damage to the nervous system. Baune et al.'s study found that in elderly patients, higher concentrations of IL-6 with chronic low-level systemic inflammation may be associated with increased mortality (34). The aging process strengthens the chronic immune response in the brain (leading to chronic cerebrovascular changes, such as amyloid protein deposition, resulting in increased leukocytes), which may be linked to an ongoing dysfunction in the central nervous system and degeneration (26, 35). Large cohort studies have demonstrated that men and older patients with moderate/severe TBI have worse long-term outcomes (35).

Lv et al. (31) developed a nomogram that could predict mortality by cooperating with COP, neurological pathogenesis, and other useful scores for neurological patients. The proposed prediction model was more accurate than the commonly used mortality scales.

Clinical nomogram models were used to examine the relationship between baseline health status and future outcomes. Physicians may be able to make informed decisions about their patients' care by incorporating clinical factors and scoring systems into user-friendly nomograms. Due to its visual appeal, intuitive nature, and appreciable functions, the nomogram has gradually gained acceptance and consolidated use in clinics to facilitate prediction and decision-making (10).

This research used the public database called MIMIC-IV, which contained a large dataset of critically ill patients, providing strong evidence for the results. Several publications have investigated TBI's incidence, mortality, risk factors, and outcomes over the past few years. The current research generated groundbreaking results. The proposed nomogram showed considerable clinical utility for predicting mortality in patients with TBI. These factors selected for the model made it convenient and straightforward to utilize to estimate mortality rates. The complexity of clinical algorithms was reduced, and a method for predicting critical illness of TBI using easily obtainable clinical variables was obtained. Secondly, compared to other commonly used scales, the proposed model's scoring system was better in comprehensively reflecting patients' overall situation with higher accuracy. It also had a higher comparison level, repeatability, and accuracy than other models, enhancing the prediction performance. By intervening early in the variables positively related to mortality, patient mortality can be reduced in clinical practice, especially for individualized therapy. Factors that contribute to death risk that cannot be changed can significantly indicate the patient's death risk. Therefore, this study can identify high-risk patients early.

This research has some limitations. Technical limitations prevented us from grading patients' imaging examination pictures, determining brain trauma degrees, or verifying patients' mental states; surgical method, surgical time, imaging information, and other scores were not included in our study. Although these variables were not included, we conducted statistical validation and found that the predictive performance of this nomogram was relatively excellent. In the near future, we plan to conduct further prospective research in the hospital where we work, including the important variables mentioned above. Future studies may be able to verify and analyze the relative data of TBI patients at the mentioned hospital through subsequent experiments. The relative data of TBI patients in the dataset may be further validated and analyzed.

## 5. Conclusion

This study achieved significant potential for TBI patients in ICU with clinical utility. The proposed TBI-IHM nomogram could estimate the mortality risk for each TBI patient. This nomogram can assist clinical doctors in identifying and making scientific clinical decisions at an early stage of TBI.

## Data availability statement

The data analyzed in this study was obtained from the Medical Information Mart for Intensive Care IV (MIMIC-IV) database, the following licenses/restrictions apply: To access the files, users must be credentialed users, complete the required training (CITI Data or Specimens Only Research) and sign the data use agreement for the project. Requests to access these datasets should be directed to PhysioNet, https://physionet.org/, 10.13026/6mm1-ek67.

## Ethics statement

The participants signed consent to complete the training course (CITI Data or Specimens Only Research, PhysioNet Credentialed Health Data Use Agreement) of the National Institutes of Health on the internet. Written informed consent for participation was not required for this study in accordance with the national legislation and the institutional requirements.

## Author contributions

GJ: data curation. LZ: funding acquisition. BM and FT: investigation. LP: project administration. LZ, KQ, and NG: resources. DL and GJ: data merging and statistical analysis. JC: writing—original draft. WH: writing—review and editing. All authors contributed to the article and approved the submitted version.
